# A new failure mechanism of electromigration by surface diffusion of Sn on Ni and Cu metallization in microbumps

**DOI:** 10.1038/s41598-018-23809-1

**Published:** 2018-04-12

**Authors:** Yuan-Wei Chang, Chia-chia Hu, Hsin-Ying Peng, Yu-Chun Liang, Chih Chen, Tao-chih Chang, Chau-Jie Zhan, Jing-Ye Juang

**Affiliations:** 1 Department of Materials Science and Engineering, National Chiao Tung University, Hsin-chu, 30010 Taiwan Republic of China; 2 Electronic and Optoelectronic System Research Laboratories, Industrial Technology Research Institute, Hsin-chu, Taiwan 31040 Republic of China

## Abstract

Microbumps in three-dimensional integrated circuit now becomes essential technology to reach higher packaging density. However, the small volume of microbumps dramatically changes the characteristics from the flip-chip (FC) solder joints. For a 20 *µ*m diameter microbump, the cross-section area and the volume are only 1/25 and 1/125 of a 100 *µ*m diameter FC joint. The small area significantly enlarges the current density although the current crowding effect was reduced at the same time. The small volume of solder can be fully transformed into the intermetallic compounds (IMCs) very easily, and the IMCs are usually stronger under electromigration (EM). These result in the thoroughly change of the EM failure mechanism in microbumps. In this study, microbumps with two different diameter and flip-chip joints were EM tested. A new failure mechanism was found obviously in microbumps, which is the surface diffusion of Sn. Under EM testing, Sn atoms tend to migrate along the surface to the circumference of Ni and Cu metallization to form Ni_3_Sn_4_ and Cu_3_Sn IMCs respectively. When the Sn diffuses away, necking or serious void formation occurs in the solder, which weakens the electrical and mechanical properties of the microbumps. Theoretic calculation indicates that this failure mode will become even significantly for the microbumps with smaller dimensions than the 18* µ*m microbumps.

## Introduction

Microbumps in three-dimensional integrated circuit (3D-IC) have emerged as a critical technology for the demand of better performance and higher packaging density^1–4^. Since the shrinkage of Si devices does not catch up with the Moore’s law, the 3D-IC technology provides an alternative way to maintain the increasing rate of packaging density^2,4,5^. Microbumps have been adopted as interconnects between chips, and they are fabricated through a transient liquid phase reaction. However, the contact area of a microbump is only 1/25 of a flip-chip (FC) bump. With the reduction of contact area, the current density flowing in a microbump becomes much larger than that in a conventional FC bump. For a 20 *µ*m microbump applied by 0.2 A, the current density reaches 6.37 × 10^4^ A/cm^2^, which is far above the threshold value of Sn electromigration (EM)^6–8^. Furthermore, the volume of a microbump is smaller than that of a FC bump by two orders, which may differ the EM failure mechanism in microbumps from the reported mechanisms in FC bumps. There were two main EM failure mechanisms reported^9,10^: the void propagation along the IMC/solder interface^11–14^ and the dissolution of under-bump-metallization (UBM)^15–20^. Both of them were correlated with the current crowding effect in the FC bumps. The small amount of solder in microbumps did not only reduce the current crowding effect^21,22^, it also could be fully transformed into the IMC in a short time. These characteristics significantly elongated the EM failure and caused the microbumps immortal in some specific test conditions^23–25^. Therefore, it is important to understand the EM failure mechanism inside microbumps. Some researchers have reported that surface diffusion of Sn occurred in microbumps during transient liquid phase reactions^26,27^. This is a very important phenomenon because the surface diffusion of Sn on Ni or Cu is much faster than the grain boundary (GB) diffusion and the lattice diffusion. Bokshteyn *et al*. reported the surface diffusion of Sn on Ni was 8 and 10 orders faster than the GB diffusion and the lattice diffusion at high temperature (700 °C)^28,29^. Besides, the diffusion was significantly enhanced by the EM^30^. However, there is no study yet to investigate EM failure in solder state in microbumps caused by the surface diffusion of Sn on the circumference of Ni and Cu UBMs. In this study, we applied EM testing on microbumps with different diameters and FC bumps. All of the UBM in these tested samples were Cu 5 *µ*m/Ni 3 *µ*m. Then a new EM failure mechanism, the surface diffusion of Sn, was found in microbumps with small volume of solder.

## Results

**Figure 1:**
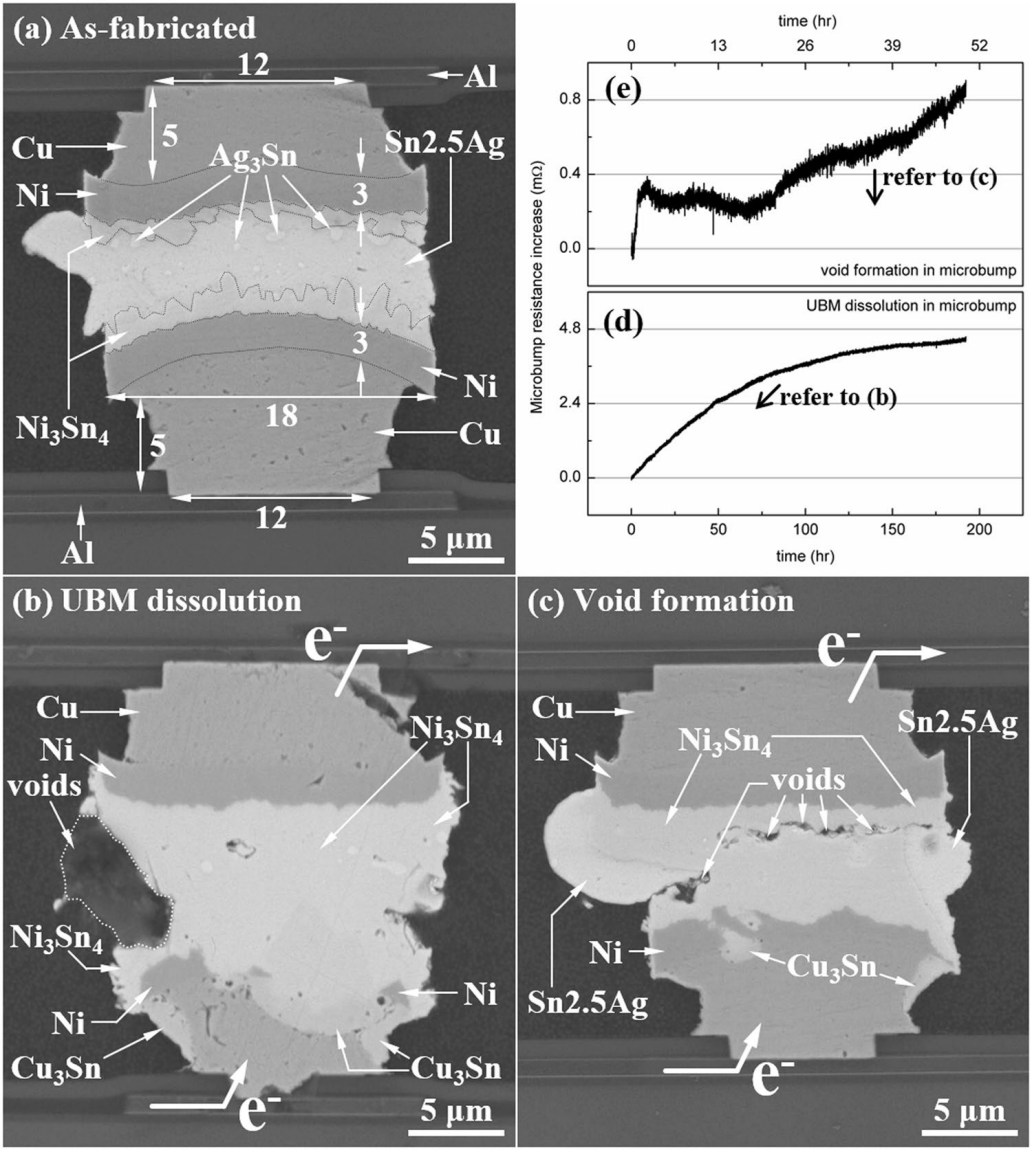
(**a**) The SEM image of the as-fabricated 18 *µ*m microbump; The SEM image showing (**b**) the UBM dissolution and (**c**) the void formation induced by EM after 9.2 × 10^4^ A/cm^2^ at 150 °C for 192.3 and 50.0 h, respectively; The resistance increase due to (**d**) the UBM dissolution in (**b**), and (**e**) the void formation in (**e**).

An as-fabricated 18 *µ*m-diameter microbump was shown in Fig. 1(a). It showed a very important specialty of microbump. The volume of solder became very small and inferior to the volume of UBM significantly. In this case, the solder could be fully consumed and transformed into IMC in a short time, as shown in Fig. 1(b). Figure 1(b and c) showed the microbumps EM tested by 9.2 × 10^4^ A/cm^2^ at 150 °C for 192.3 and 50.0 h respectively. The IMCs including Ni_3_Sn_4_, Cu_6_Sn_5_, and Cu_3_Sn were found with much better EM resistance than the solder according to the higher critical product. The critical product indicates the ability of materials to resist EM, and it is represented as^31^

1 $$C.P.={L}_{c}\,{j}_{c}=\frac{{\sigma }_{c}\Omega }{{Z}^{\ast }e\rho }$$

where L_*c*_ is the critical length, j_*c*_ is the critical current density, *σ*_*c*_ is the stress as the critical length, Ω is the atomic volume, Z^∗^ is the effective charge number, e is the charge of electron, and *ρ* is the resistivity. If we take the stress at the elastic limit, the stress is the yield strength, *σ*_*y*_. From the parameters in Table 1, we could not find big difference between Ω, Z^∗^, and *ρ*. However, the *σ*_*c*_ of IMCs are at 45 to 57 times higher than that of solder. Accordingly, the EM resistance of all IMCs are much higher than that of solder as well. Once solder reacts with the UBM and fully transforms into IMCs, the microbump becomes an IMC joint, which can sustain under EM for a long time. That tremendously change the EM failure mechanism in microbumps rather than FC bumps.

**Table 1:** The parameters for the calculation of the critical product.

Material	σ_y_	Density (g/cm^3^)	M_avg_ (g/mol)	Ω (10^−23^ cm^3^)	Z^*^	ρ (μΩ-cm)
Sn3.5Ag	24.8 MPa^50,51^	7.4^33^	118.69	2.66 2.705 (Sn)^52^ 2.7 (Sn)^53^ 2.72 (Sn)^54^	18^30^	12.3^55^ 12.32^50^
Cu_6_Sn_5_	1.15 GPa^51^	8.28^50^	88.62	1.78 2.0^18^	68–87^56^ 50^18^ 26 (Cu)^57^ 36 (Sn)^57^	17.54^50^
Cu_3_Sn	1.41 GPa^51^	8.90^50^	77.34	1.44	27 (Cu)^57^ 24 (Sn)^57^	8.93^50^
Ni_3_Sn_4_	1.13 GPa^51^	8.65^50^	92.98	1.79	21 (Ni)^54^ 21 (Sn)^54^	28.57^50^

M_avg_: Average atomic weight; Ω = *Density/M*_*avg*_.

In the previous studies of FC bumps, there were two EM failure mechanisms reported: (1) the void propagation along the IMC/solder interface^11–14,32^ and (2) the fast UBM dissolution/IMC formation^15–20,33–35^. Due to the small volume of solder in microbump, the fast reaction between UBM and solder no longer caused the joint failure. Instead, the formation of IMC joint significantly elongated the EM failure time^23^. Moreover, the fast IMC formation suspended the void propagation as shown in Fig. 1(c). Sometimes the fast transformation was accompanied by the void formation at the IMC/solder interface. However, the volume of solder was too small, and the height of solder was too short. The full transformation from solder into IMCs was faster than the void propagation through the entire interface so the void propagation was stopped. The formation of IMC joint overtook the effect of void propagation in this case. The microbump resistances of full transformation and void propagation, shown in Fig. 1(d and e) respectively, indicates the same tendency. The full transformation from the solder into IMC (in Fig. 1(b)) induced only 4.46 mΩ of resistance increase, which was 3.72% of the total microbump resistance (119.77 mΩ). The void propagation was overtaken by the IMC formation and therefore did not cause obvious resistance increase as the void propagation in FC bumps did. Even we could already observe the discontinuous void at the entire interface, the consequent resistance increase was only 0.86 mΩ. However, a new EM failure mechanism, surface diffusion of Sn, was found in this study. It is a phenomenon that a big portion of Sn diffused along the circumference surface of UBM and than induced the open failure.

**Figure 2:**
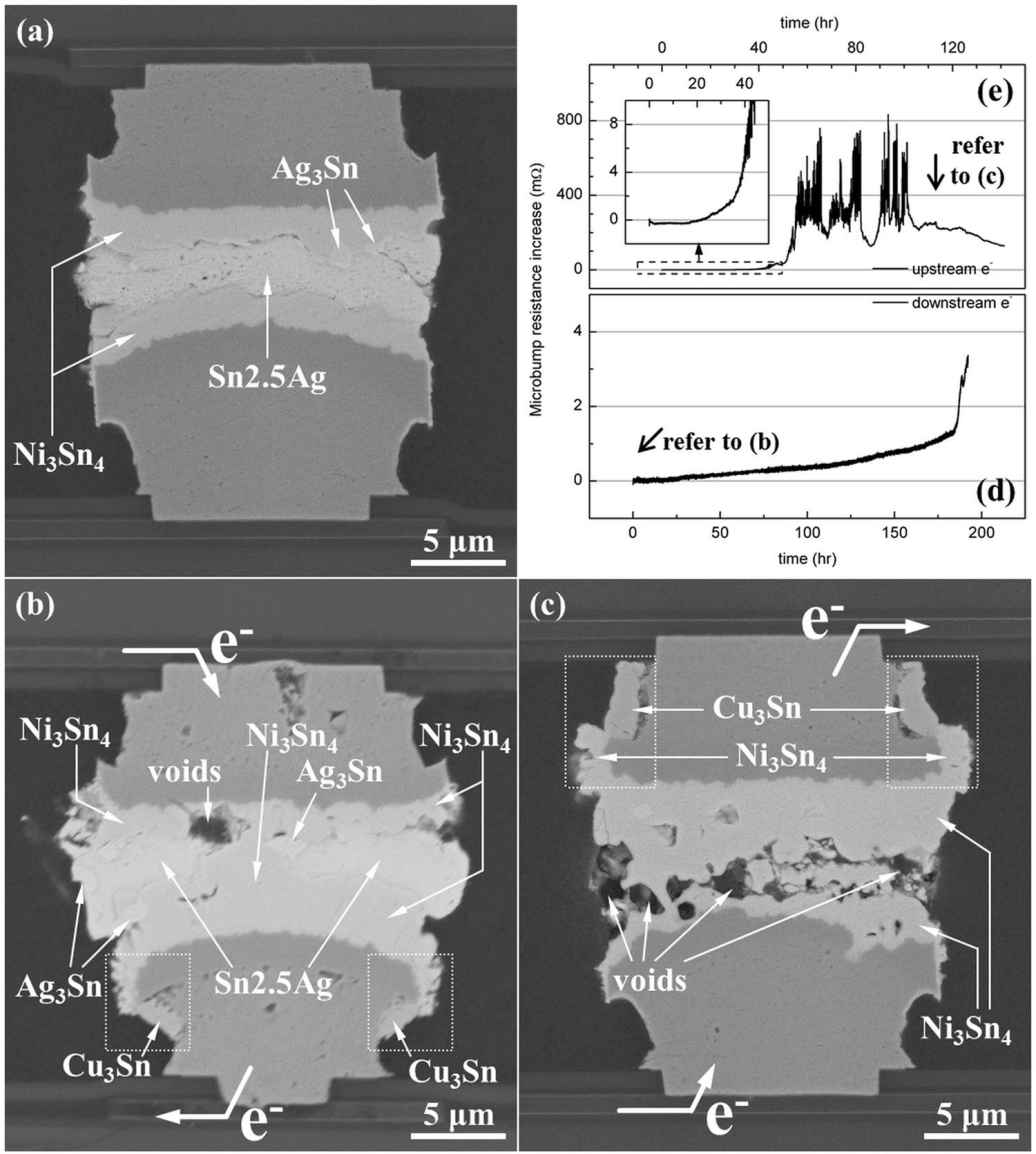
(**a**) The SEM images of the dummy 18-*µ*m microbump suffered from the same thermal history as the current stressed bumps. Only IMC thickening was observed. After EM tested under 9.2 × 10^4^A/cm^2^ at 150 °C, side diffusion took place in microbumps. (**b**) With slight side-diffusion of Sn and (**c**) With severe void formation and failure caused by side-diffusion Sn; (**d**) The resistance curve for the microbump in (**b**), and (**e**) The resistance for the microbump in (**c**), which indicates the failure under EM testing.

Comparing with the as-prepared microbump shown in Fig. 1(a), surface diffusion of Sn occurs on the circumference of the Ni/Cu UBM during the current stressing in microbumps. Figure 2(a) showed a dummy microbump suffered from the same thermal history as the current stressed microbump in Fig. 2(c). There was still 2.7 *µ*m-thick solder remained un-reacted, and the surface diffusion of Sn was not obvious in the dummy microbump without current stressing. Instead, it was significantly activated by the current stressing in the EM tested microbumps (Fig. 2(b and c)). The surface diffusion drains Sn atoms to the circumference and causes the serious void formation and necking in microbumps. Figure 2(b) presents the cross-sectional SEM image of the microbump after EM tested under 9.2 × 10^4^A/cm^2^ at 150 °C. We could observe the beginning of the surface diffusion of Sn at the circumference of the anode UBM (marked by the dashed rectangles). There were still some solder unreacted, and an obvious void was already able to be observed. The corresponding resistance (Fig. 2(d)) showed a different behavior from the resistance curves of UBM dissolution and void propagation. The resistance gradually increased in the beginning of EM testing. The growth of IMC was responsible for the lifting of microbump resistance. While the side-diffusion induced the void formation, the resistance increase immediately reacted by raising from 1.35 to 3.29 mΩ in 7.94 h. Figure 2(c) presents the cross-sectional SEM image for the microbump after the upward current stressing at 9.2 × 10^4^ A/cm^2^ for 143 h. The corresponding resistance was demonstrated in Fig. 2(e) Serious void formation was observed in this microbump. Almost all the solder were transformed into Ni_3_Sn_4_ intermetallic compounds (IMCs). It is noteworthy that significant amount of Sn diffused to the circumference of the top UBM and formed Ni_3_Sn_4_ and Cu_3_Sn IMCs there, as labeled by the dashed rectangles in the figure. On the contrary, the dummy microbump did not have obvious surface diffusion of Sn, and there was still 2.7 *µ*m-thick solder remained unreacted. Unlike the slight increase of microbump resistance induced by the UBM dissolution and void propagation, the serious void formation induced by the surface diffusion of Sn caused several hundred milli-ohm of resistance increase as shown in Fig. 2(e). Besides, the microbump resistance in the late stage of EM testing was very unstable.

**Figure 3:**
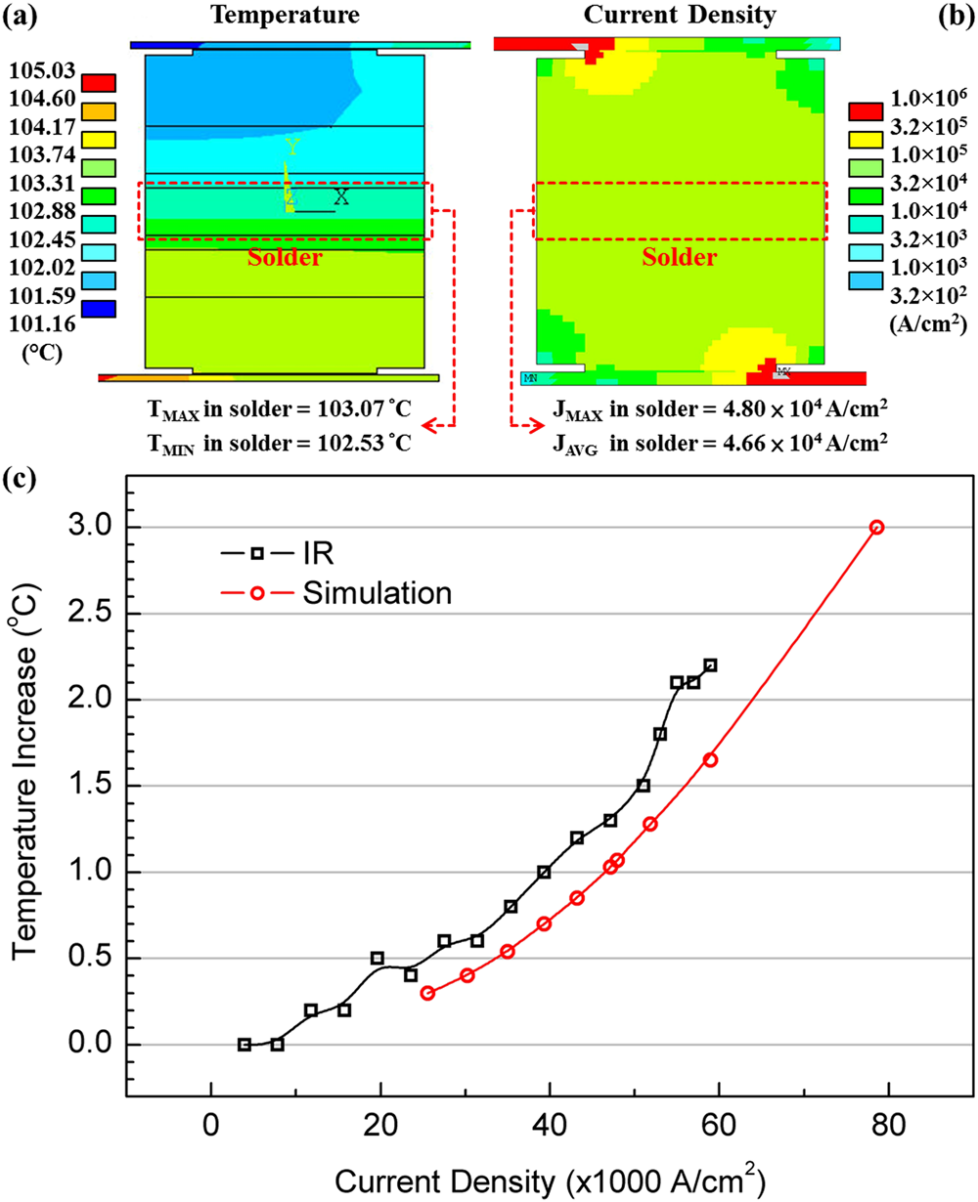
The simulated (**a**) distribution of temperature and (**b**) distribution of current density in the 18 *µ*m microbump; (**c**) Plot of solder temperature against applied current density obtained from the simulation and infrared microscopy (IR).

The surface diffusion of Sn in microbumps was a very common phenomenon; it did not only happen in the extreme testing conditions. Due to the chip-on-chip (COC) package, the heat dissipation of the tested samples were very good. Figure 3(a and c) demonstrated the cross-sectional temperature distribution in a stressing microbump and the temperature increase obtained by the simulation and the infrared microscope (IR). A 8.0 × 10^4^ A/cm^2^ current density only induced 3 °C of temperature increase as shown in Fig. 3(c). Even a 9.2 × 10^4^ A/cm^2^ current density barely induced 5 °C of temperature increase in the solder. The ambient temperature of EM testing was 150 °C. With the temperature increase induced by Joule heating, the maximum temperature of solder in the EM testing microbump did not exceed 155 °C, which should not significantly affect the failure mechanism. The cross-sectional current density distribution in the current stressing microbump was shown in Fig. 3(b). Because of the small diameter of microbumps, the difference of cross-section area between the traces and the microbumps was not big. Therefore, the current crowding effect is very slight in the microbumps^21,22^. As shown in Fig. 3(b), the maximum current density was only 4.80 × 10^4^ A/cm^2^ when the average one was 4.66 × 10^4^ A/cm^2^. However, the necessary conditions to activate the surface diffusion of Sn were still not clear. The surface diffusion of Sn occurred on the circumference of both the cathode and anode UBM. The present observation only implies that the EM is an important activation because the surface diffusion of Sn was not found in most of the dummy microbumps. More study is required on this topics.

**Figure 4:**
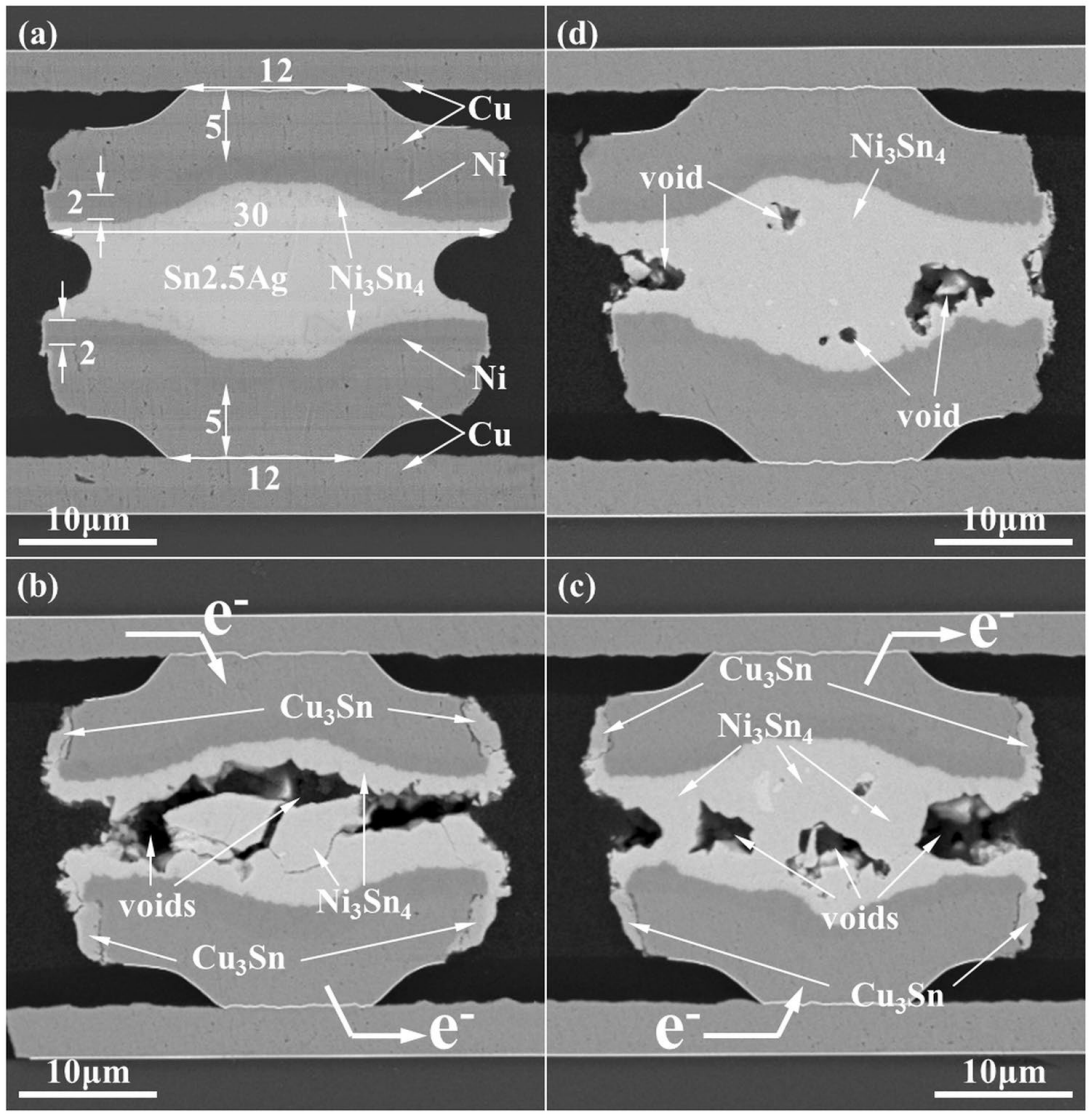
(**a**) The SEM image of the as-prepared microbump with 30 *µ*m in diameter; (**b**) and (**c**) the SEM image of microbumps showing severe void formation or necking after the EM test by 4.0 × 10^4^ A/cm^2^ at 150 °C for 2320 h; (**d**) the SEM image of the dummy microbump with the same thermal history as the stressed microbumps.

The surface diffusion of Sn appears to be a universal EM reliability issues, and similar phenomena occurred in the microbumps with 30 *µ*m in diameter. Figure 4(a) presents the cross-sectional SEM image for the 30 *µ*m microbump before EM tests. The UBM structure was 5 *µ*m-Cu/2 *µ*m-Ni and the solder height approximately 5.7 *µ*m. Daisy chain structures of 40 microbumps were adopted for EM tests. After current stressing by 4.0 × 10^4^ A/cm^2^ at 150 °C for 2320 h, severe void formation occurred in some of the microbumps. Figure 4(b) presents the worst microbump with almost open failure. The electrons drifted downward in this bump. All the SnAg solders between the top and between UBMs were transformed into Ni_3_Sn_4_ IMC. The surface diffusion of Sn took place in both top and the bottom circumference of the Cu UBMs. The Sn atoms also reacted with the Ni and Cu to form Ni_3_Sn_4_ and Cu_3_Sn IMCs, respectively. This is because there was more Cu than Sn on the side wall of the Cu UBM. Therefore Cu_3_Sn formed instead of Cu_6_Sn_5_ IMCs. Diffusion of Sn on the circumference of Ni and Cu also occurred in other microbumps in the daisy chains. Figure 4(c) present another microbump having the surface diffusion of Sn and serious void formation. The microbump in Fig. 4(c) had the surface diffusion of Sn on both the top and bottom Cu UBMs. Serious necking was observed on the right side of the microbumps. On the contrary, the surface diffusion was not found in the dummy microbump in Fig. 4(d). Although some voids were also observed in the dummy microbump, it was obvious the microbump became an IMC bump and lasted for longer. The open failure caused by big voids (Fig. 4(b)) and the necking of joint (Fig. 4(c)) could not be found in the dummy microbumps.

**Figure 5:**
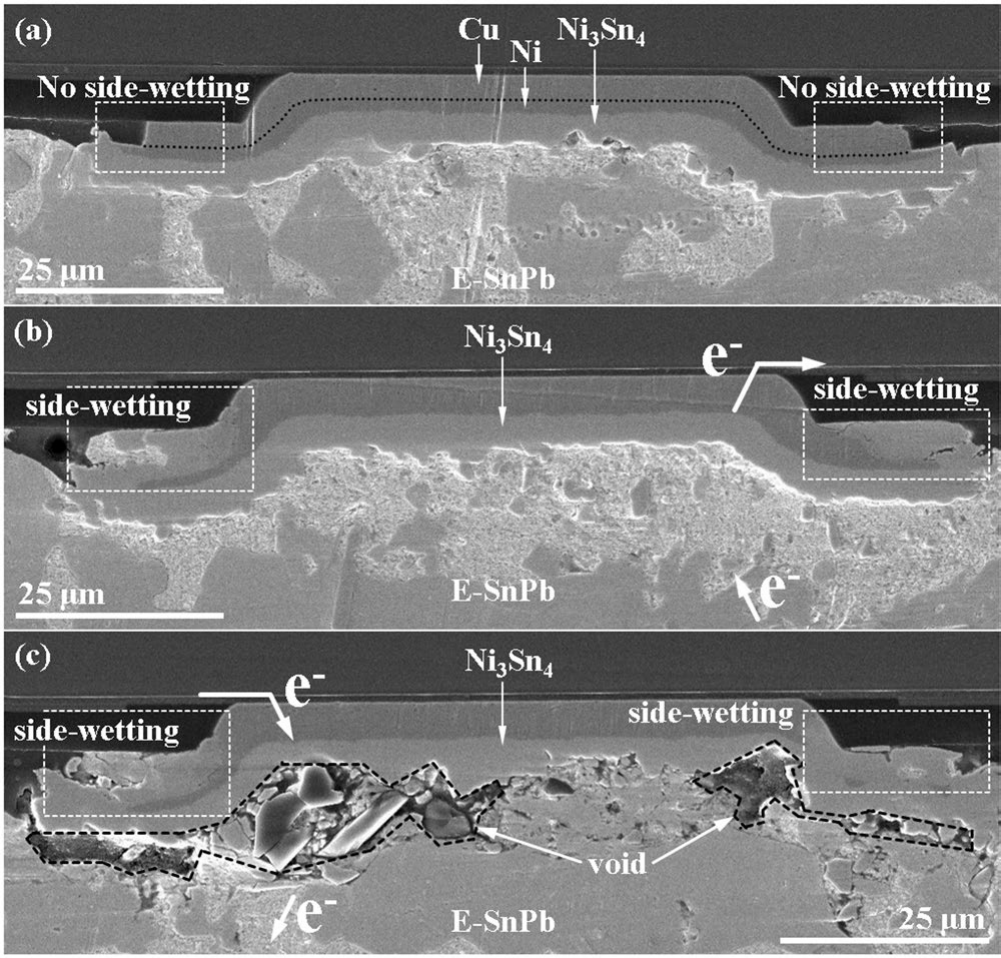
(**a**) The SEM image of the dummy 120-*µ*m FC solder joints suffered from the same thermal history as the current-stressed joints. The SEM image showing the microstructure for the joint stressed by (**b**) an upward and (**c**) a downward electron flow for 1087 h.

Actually, the surface diffusion of Sn (solder) induced by EM could also be found in the 120 *µ*m-diameter FC solder joint as shown in Fig. 5. The dashed rectangles in Fig. 5 indicated the UBM circumference of FC joints. The dummy joints in Fig. 5(a) suffered from the same thermal history as the current stressing joints, and no surface diffusion of Sn was found in the dummy ones. On the contrary, the surface diffusion of Sn were significant in both of the FC joints tested by the upward and downward electron flow as shown in Fig. 5(b and c) respectively. However, the failure was mainly caused by the void propagation along the IMC/solder interface. The surface diffusion of Sn did not play an important role in the EM failure mechanism in the FC solder joints. As a result, it is very obvious that the small volume of Sn (solder) emphasized the importance of surface diffusion effect.

## Discussion

The surface diffusion of Sn emerges to be the main EM failure mechanism in microbumps in this study. This is because the solder volume is very small, and once some solders are drawn away, voids form in the microbumps. Interestingly, the surface diffusion of Sn was only observed in current stressed microbumps and flip-chip joints as shown in Figs 2, 4, and 5. Sometimes the surface diffusion was only found at the anode side (Fig. 2(b and c)); sometimes it was found at both of the anode and cathode side. Because the metal atoms was driven by the electronic wind force during the EM testing^31^, it is reasonable that the surface diffusion appeared at the anode side. The surface diffusion at cathode side might be induced by the back stress in the solder generated by electron flow^36^. The electron flow drove atom from cathode end to the anode end, and the back stress would increase the stress in the solders and try to push the Sn atoms back to cathode. It is speculated that at some locations in the cathode end of solders, the stress may be high enough to trigger the Sn atoms in the periphery of the microbumps to move to the side walls of the UBMs. Therefore, we occasionally observed side diffusion in the cathode end. Taking Ni for example, the surface diffusion coefficient is higher than the bulk and grain boundary diffusion by 10 and 8 orders at 700 °C^28,29^. No matter of the surface diffusion at the anode or cathode side, current stressing was the most important factor that triggered the surface diffusion. The surface diffusion of Sn was only observed in the current stressed microbump or flip-chip joints.

In the following paragraphs, we will calculate the volume of Sn diffusion to the side walls of Ni and Cu UBMs. In the microbump in Fig. 2(c), the average thickness of the voids was measured to be 2.0 *µ*m. The diameter of the microbump is 18 *µ*m, so the volume of the voids was estimated to be 514.0 *µ*m^3^. On the other hand, the original volume of the solder was calculated to be 1577.0 *µ*m^3^. It is noteworthy that the formation of Ni_3_Sn_4_ IMCs induces 10.7 and 7.1% of volume shrinkage when the Ni_3_Sn_4_ and Cu_3_Sn are forming, as depicted in Table 2. The volume shrinkage may cause voids with volume of 168.8 *µ*m^3^ (=1577 *µ*m^3^ × 10.7%) if the solder is fully reacted into Ni_3_Sn_4_. Therefore, approximate Sn volume of 345.2 *µ*m^3^ (=514–168.8 *µ*m^3^) was induced by the surface diffusion of Sn to the circumference of the Cu and Ni UBMs.

**Table 2:** The volume ratio during the following reactions: 3 Cu + Sn → Cu_3_Sn and 3 Ni + 4 Sn → Ni_3_Sn_4_.

Material	Molecular Weight (g/mol)	Density (g/cm^3^)	Normalized volume (%)
Cu	63.6	8.96	56.9
Sn	118.76	7.37	43.1
Cu_3_Sn	309.4	8.90	92.9
Ni	58.7	8.91	23.5
Sn	118.76	7.37	76.5
Ni_3_Sn_4_	650.9	8.65	89.3

The normalized volume is the volume of elements (compounds) normalized to the initial total volume.

We will calculate the depleted solder volume due to the surface diffusion of Sn using Fig. 2(c). As indicated in the figure, Sn atoms diffused to the circumference of the top Ni and Cu UBMs, and they reacted with Ni and Cu to form Ni_3_Sn_4_ and Cu_3_Sn, respectively. The thickness of the Ni_3_Sn_4_ and Cu_3_Sn was measured to be 1.5 *µ*m and 1.5 *µ*m, respectively. The height of the Ni_3_Sn_4_ and Cu_3_Sn was 2.5 *µ*m and 4.0 *µ*m, respectively. We assumed the thickness of the Ni_3_Sn_4_ is the same of every location of the circumference of the Ni UBM, and the thickness of the Cu_3_Sn is also the same at everywhere of the periphery of the Cu UBM. The consumed volume of Sn can be calculated using the volume ratio of Ni_3_Sn_4_ and Cu_3_Sn in Table 2. The Sn volume consumed by the Ni_3_Sn_4_ and Cu_3_Sn formation is 135.3 *µ*m^3^ and 186.3 *µ*m^3^, respectively. The sum of these two values yields 322.2 *µ*m^3^ (=186.7 + 135.5 *µ*m^3^), which is close to the void volume of 345.2 *µ*m^3^ in the previous paragraph. Therefore, surface diffusion of Sn serves the main reason causing the void formation in the microbumps during the EM.

**Figure 6:**
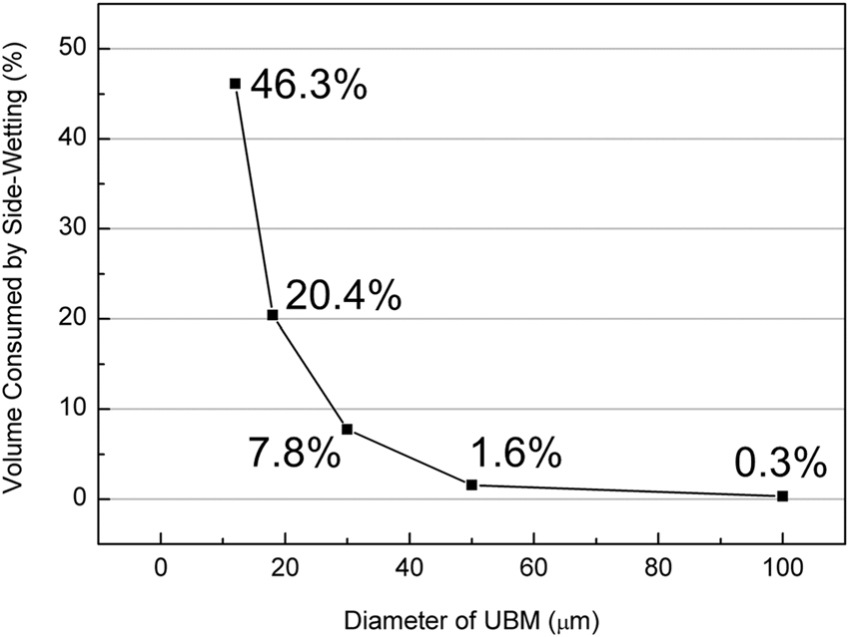
The volume percentage of depleted solder due to the surface diffusion of Sn as a function of UBM diameter.

It is noteworthy that the Sn atoms also diffused to the circumference of the UBM in traditional FC solder joints as shown in Fig. 5(b and c)^9,37–41^. However, no necking or void formation caused by the out diffusion of Sn atoms. This is because these are plenty of Sn in FC solder joints. For a typical FC joint with a diameter of 100 *µ*m and a bump height of 80 *µ*m, the total solder volume was estimated to be 6.3 × 10^5^ *µ*m^3^ as shown in Table 3. If we assume the UBMs consists of 5 *µ*m Cu and 3 *µ*m Ni, and Sn atoms also diffuse to the side walls of Cu and Ni UBMs to form Cu_3_Sn and Ni_3_Sn_4_. The depleted Sn volume is 852.6 *µ*m^3^ and 1014.3 *µ*m^3^ due to the formation of Cu_3_Sn and Ni_3_Sn_4_ respectively, resulting in only 0.3% of depletion of solder volume. Therefore, no necking or serious void formation due to the surface diffusion of Sn atoms. Nevertheless, the volume percentage of the surface diffusion increases as the dimension of the solder joint decreases. Table 3 lists the depletion percentage for the solder joints with 100 *µ*m, 50 *µ*m, 30 *µ*m, 18 *µ*m, and 12 *µ*m. The calculation demonstrates that the depletion volume percentage is 0.3%, 1.6%, 7.8%, 20.4%, 46.3%, respectively. Figure 6 presents the percentage of Sn consumed by the surface diffusion as a function of the diameter of the UBM. The calculation assume the UBM is 5 *µ*m-thick Cu/3 *µ*m-thick Ni for all the solder joints in Table 3. It is noted that the thickness of the UBM cannot be reduced because of the reliability concern of metallurgical reactions. If the UBM is too thin, spalling of IMCs may occur during reflow^12,42–45^. Or the UBM will dissolve completely in the beginning stage of EM testing^46,47^. In the present study, the out diffusion of Sn atoms depletes 20.4% (322.2 *µ*m^3^) of the solder in the microbumps with 18 *µ*m in diameter. As the dimension shrinks down to 12 *µ*m in diameter, the surface diffusion of Sn will deplete 46.3% of the solder in the microbumps. Therefore, surface diffusion of Sn will be a critical issue for EM in microbumps, and it may dominate the failure mode of EM.

**Table 3:** The volume percentage of solder depleted by surface diffusion of Sn.

D_UBM_ (μm)	h_so_ (μm)	V_so_ (μm^3^)	V_NS_ (μm^3^)	V^1^_so_ (μm^3^)	V_CS_ (μm^3^)	V^2^_so_ (μm^3^)	V_sd_ (%)
12.0	4.0	4.5 × 10^2^	147.3	126.2	179.1	83.1	46.3
18.0	6.2	1.6 × 10^3^	217.9	186.7	292.2	135.5	20.4
30.0	10.0	7.1 × 10^3^	359.3	307.8	518.4	240.5	7.8
50.0	30.0	5.9 × 10^4^	594.9	509.7	895.4	415.4	1.6
100.0	80.0	6.3 × 10^5^	1184.0	1014.3	1837.8	852.6	0.3

D_UBM_: the diameter of UBM.

h_so_: the height of solder.

V_so_: the total volume of soler.

V_NS_: the volume of Ni_3_Sn_4_ on the circumference of Ni UBM.

V^1^_so_: the solder consumed by the Ni_3_Sn_4_. V^1^_so_ = V_NS_ × 76.5/89.3.

V_CS_: the volume of Cu_3_Sn on the circumference of Cu UBM.

V^2^_so_: the solder consumed by the Cu_3_Sn. V^2^_so_ = V_CS_ × 43.1/92.9.

V_sd_: the solder consumed by the surface diffusion. V_sd_ = (V^1^_so_ + V^2^_so_)/V_so._

By the surface diffusion shown in Fig. 2(c), a minimum diffusion coefficient was obtainable. The diffusion length from the geometric center of depleted Sn to the circumference is 13 *µ*m, and the diffusion took 53.5 h to achieve (the timing that the resistance suddenly raised). The diffusion velocity equals 13 *µ*m/53.5 h, and that is 6.75 × 10^−9^ cm/s. The diffusion flux, J, can be represented as:

2 $$J=C\upsilon $$

where C is the concentration and *υ* is the diffusion velocity. The flux induced by EM, J_*EM*_, is^31^

3 $${J}_{em}=C\frac{D}{kT}{Z}^{\ast }e\rho j$$

where D is the diffusion coefficient, k is Boltzmann’s constant, T is the temperature, Z^∗^ is the effective charge number, e is the electron charge, *ρ* is the resistivity, and j is the applied current density. By combining Eq. 2 and Eq. 3, we obtain

4 $$D=\frac{\upsilon kT}{{Z}^{\ast }e\rho j}$$

Considering the Joule heating, the temperature of Sn was 155 °C (428 K) under 9.2 × 10^4^ A/cm^2^ of current stressing. According to previous study, Z^∗^ of Sn is 17^48^. The resistivity, *ρ*, of Sn3.5Ag is 12.5 *µ*Ω-cm Therefore, the diffusion coefficient, D, was calculated as 1.27 × 10^−11^ cm^2^/s. In this calculation, the *υ* was underestimated because the sudden resistance change could only detect the timing of void formation induced by surface diffusion. However, the beginning of surface diffusion was not achievable. To obtain the diffusion velocity, we took the beginning of EM testing as the beginning of surface diffusion. It is highly possible for us to overestimate the total time of surface diffusion and therefore underestimate the diffusion velocity and the diffusion coefficient as well. In previous study, the diffusion coefficient of Sn to diffuse in Ni was 2.65 × 10^−12^ and 3.73 × 10^−12^ cm^2^/s at 160 and 180 °C^49^. The self diffusion coefficient was 4.7 × 10^−15^ cm^2^/s at 150 °C^32^. The value we obtained by surface diffusion was higher than the values in previous study by 1 to 3 orders, which agreed with the previous study^28,29^. The fast surface diffusion of Sn caused a new EM failure mechanism, which may be more important than the UBM dissolution and void propagation in the microbumps with tiny volume of solder.

## Conclusions

In summary, we observed a new EM failure mechanism in microbumps, surface diffusion of Sn. Sn atoms diffused along the surface of the circumferences of the Ni and the Cu UBMs during EM testing, and formed Ni_3_Sn_4_ and Cu_3_Sn, respectively. Unlike the slight resistance changes induced by UBM dissolution and void propagation, the void induced by the surface diffusion of Sn caused several hundred milli-ohm of resistance increase. Although the necessary condition to activate the surface diffusion of Sn was unclear, the EM was definitely a very important factor. Besides, the surface diffusion of Sn also happened in FC solder joints but did not play an important role because of the large solder volume. The small volume of Sn emphasized the importance of surface diffusion. The depletion percentage of the Sn volume was calculated to be approximately 20.4% of the original solder volume of the microbump with 18 *µ*m in diameter. In addition, the depletion of the Sn caused serious void formation or necking in the microbumps, resulting in deteriorating electrical and mechanical properties. This failure mode will become even significantly for the microbumps with smaller dimensions than the 18 *µ*m microbumps. According to our calculation, the surface diffusion coefficient of Sn should be higher than 1.27 × 10^−11^ cm^2^/s, which exceeded the value of Sn diffusion in Ni by several orders. Therefore, it is urgent to figure out the solution to the surface diffusion of Sn atoms onto the circumferences of the UBMs.

## Methods

Microbumps of Sn2.5Ag with Cu/Ni UBM on both chip side and interposer side were selected for electromigration tests. Two kinds of microbump were adopted for EM tests: 18 *µ*m and 30 *µ*m diameter. The structures were vertically symmetric: the dimensions and materials of UBMs on the chip side and on the interposer side were the same. Figure 1(a) present the cross-sectional SEM image of the microbump with 18 *µ*m in diameter before current stressing. The thickness of Al pad was 0.8 *µ*m. The UBM was consisted of 100 nm thick Ti adhesive layer, 5.0 *µ*m thick electroplated Cu, and 3.0 *µ*m thick electroplated Ni. The Sn2.5Ag solder was made by electroplating, too. In the 18 *µ*m microbump, the diameter of Al pad, passivation opening, and UBM opening were 20 *µ*m, 12 *µ*m, and 18 *µ*m respectively. In the 30 *µ*m microbump, they were 30 *µ*m, 18 *µ*m, and 30 *µ*m respectively. The microbumps were bonded by thermo-compression at 280 °C for a few seconds. After thermo-compression, the solder was 6.2 *µ*m in height in the 18 *µ*m microbump, and the Ni_3_Sn_4_ IMCs were about 1.0 *µ*m thick on both the top and the bottom interfaces. Some dispersed Ag_3_Sn particles were observed in the Sn2.5Ag solder. Yet the total thickness of the UBM layer was 16 *µ*m, thus the volume of the UBMs is larger than that of solder by 2.5 times. For traditional FC solder joints, the volume of solder is much larger than the volume of the UBMs as shown in Fig. 5. The diameter of passivation opening and UBM opening were 85 and 120 *µ*m in the FC joints. This difference is expected to affect the EM behavior in the microbump significantly. The structure for the microbumps with 30 *µ*m in diameter was shown in Fig. 4(a).

The applied current density in the 18 *µ*m microbumps, the 30 *µ*m microbumps, and the FC joints were 9.2 × 10^4^, 4.0 × 10^4^, and 7.1 × 10^3^ A/cm^2^. The EM tests were performed on a 150 °C hotplate. After certain periods of EM testing, the current stressing was terminated and the tested samples were polished for microstructure analysis using a SEM. The 18 µm-diameter microbumps were also subjected to EM tests by 4.6 × 10^4^ A/cm^2^. However, we did not observe void formation due to the side diffusion of Sn in these microbumps. This may be attributed to the following reason: The length of trace in the 18 µm-microbump samples is shorter than that of the 30 µm-microbump samples. Therefore, the Joule heating effect induced 18 µm-diameter microbumps is lower, resulting no obvious EM effect at this stressing condition. The compositional simulation was performed by finite-element modeling.
